# Supplementary material for: Germline Variant Call Accuracy in Whole Genome Sequence Data from Canine Formalin-Fixed Paraffin-Embedded Tissue Samples
Source: Genes (Basel). 2025 Nov 11;16(11):1371. doi: 10.3390/genes16111371 (PMC12652997; doi:10.3390/genes16111371)
Supplement: Supplementary file 1 [file genes-16-01371-s001.zip › Figure_S1_IGV_screenshots.pdf]

Figure S1. Comparison of WGS data of three dogs derived from fresh frozen (FF) EDTA blood versus formalin-fixed paraffin-embedded (FFPE) spleen tissue. The figure shows IGV screenshots from two different 25 kb chromosomal regions on chromosome 5 and 17 illustrating overall consistency between FF and FFPE data with some exceptions, notably in extremely GC-rich regions. The region on chromosome 5 harbors the *EFNB3* gene and causal variant for congenital mirror movement disorder 1 (CMM1). The region on chromosome 17 harbors the *FOXI3* gene containing some extremely GC-rich sequences. The following sub-regions are shown in detail:

Interval (UU_Cfam_GSD_1.0)	Length	GC content	Remarks
Chr5:32,790,001-32,815,000	25 kb	56.7%	
Chr5:32,799,001-32,799,500	500 bp	61.2%	*EFNB3*, exon 4
Chr5:32,799,501-32,800,000	500 bp	69.2%	*EFNB3*, exon 5, causal variant for CMM1
Chr17:38,300,001-38,325,000	25 kb	45.3%	
Chr17:38,315,501-38,316,500	1000 bp	83.8%	*FOXI3*, promoter and partial exon 1
Chr17:38,319,101-38,319,600	500 bp	54.2%	*FOXI3*, partial exon 2

(**a**) Dog #1, chr 5
(**b**) Dog #2, chr 5
(**c**) Dog #3, chr 5
(**d**) Dog #1, chr 17
(**e**) Dog #2, chr 17
(**f**) Dog #3, chr 17

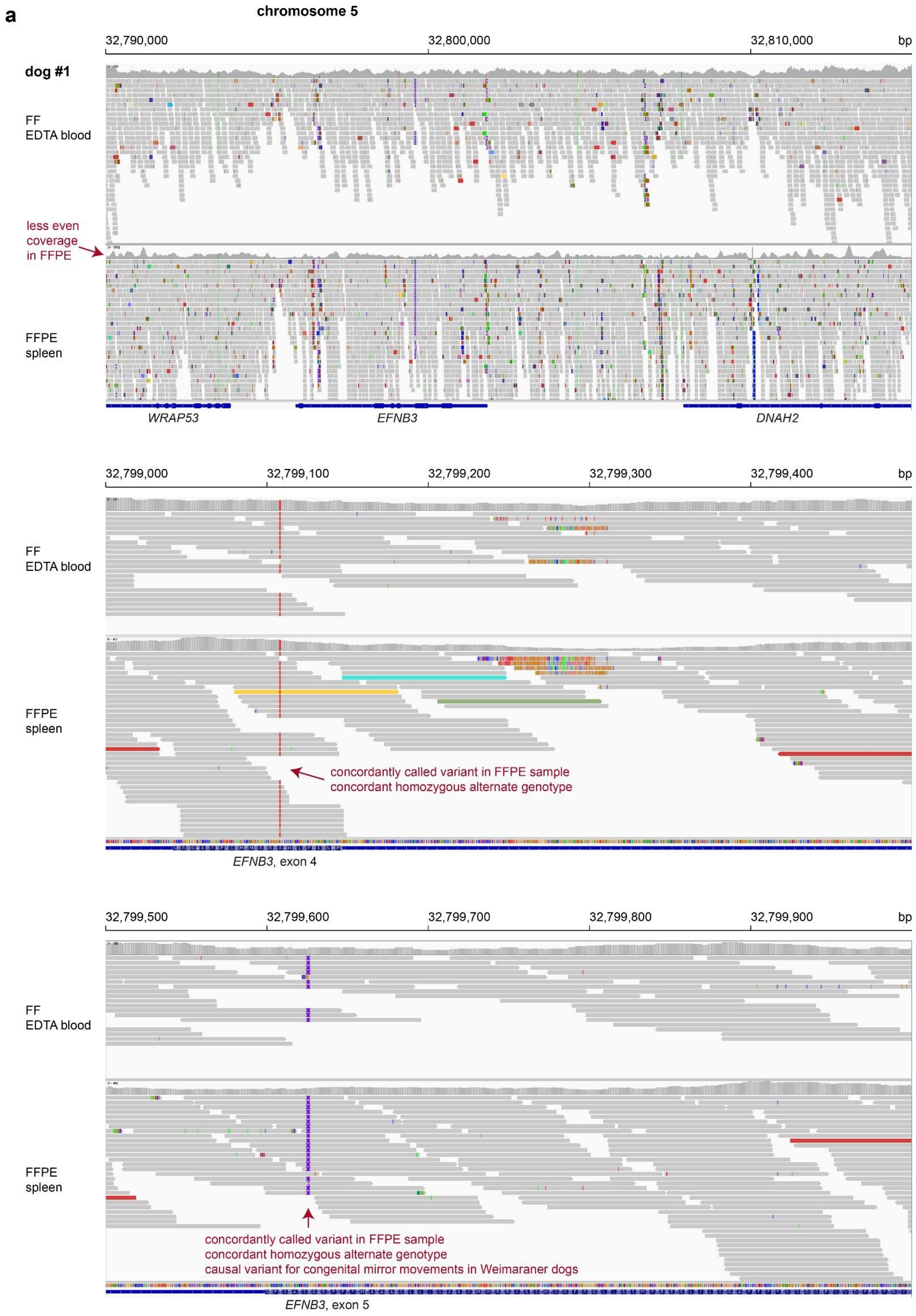
a
chromosome 5
32,790,000
32,800,000
32,810,000
bp
dog #1
FF
EDTA blood
less even coverage in FFPE
FFPE
spleen
WRAP53
EFNB3
DNAH2
32,799,000
32,799,100
32,799,200
32,799,300
32,799,400
bp
FF
EDTA blood
FFPE
spleen
concordantly called variant in FFPE sample
concordant homozygous alternate genotype
EFNB3, exon 4
32,799,500
32,799,600
32,799,700
32,799,800
32,799,900
bp
FF
EDTA blood
FFPE
spleen
concordantly called variant in FFPE sample
concordant homozygous alternate genotype
causal variant for congenital mirror movements in Weimaraner dogs
EFNB3, exon 5

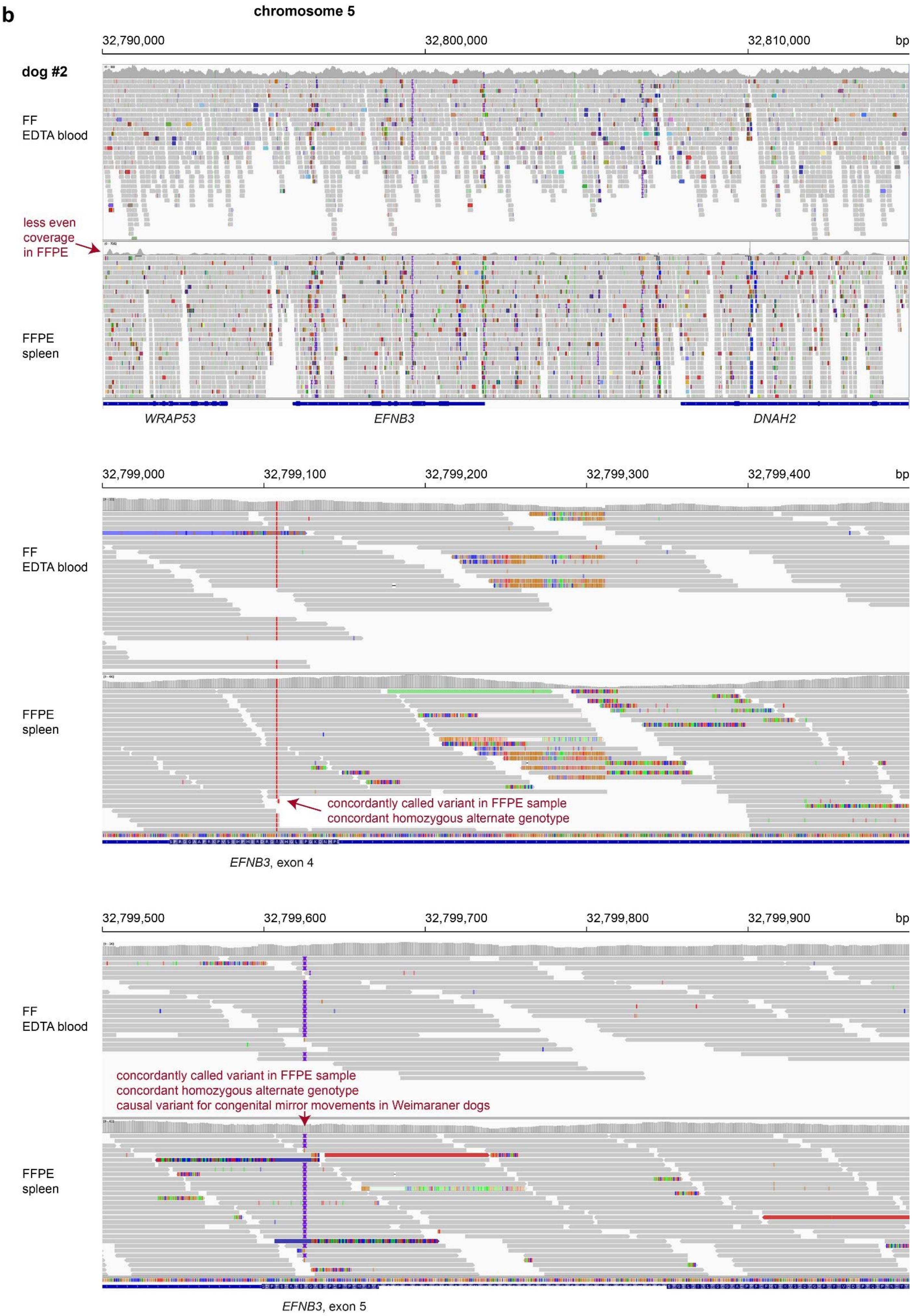
b
chromosome 5
32,790,000
32,800,000
32,810,000
bp
dog #2
FF
EDTA blood
less even
coverage
in FFPE
FFPE
spleen
WRAP53
EFNB3
DNAH2
32,799,000
32,799,100
32,799,200
32,799,300
32,799,400
bp
FF
EDTA blood
FFPE
spleen
concordantly called variant in FFPE sample
concordant homozygous alternate genotype
EFNB3, exon 4
32,799,500
32,799,600
32,799,700
32,799,800
32,799,900
bp
FF
EDTA blood
concordantly called variant in FFPE sample
concordant homozygous alternate genotype
causal variant for congenital mirror movements in Weimaraner dogs
FFPE
spleen
EFNB3, exon 5

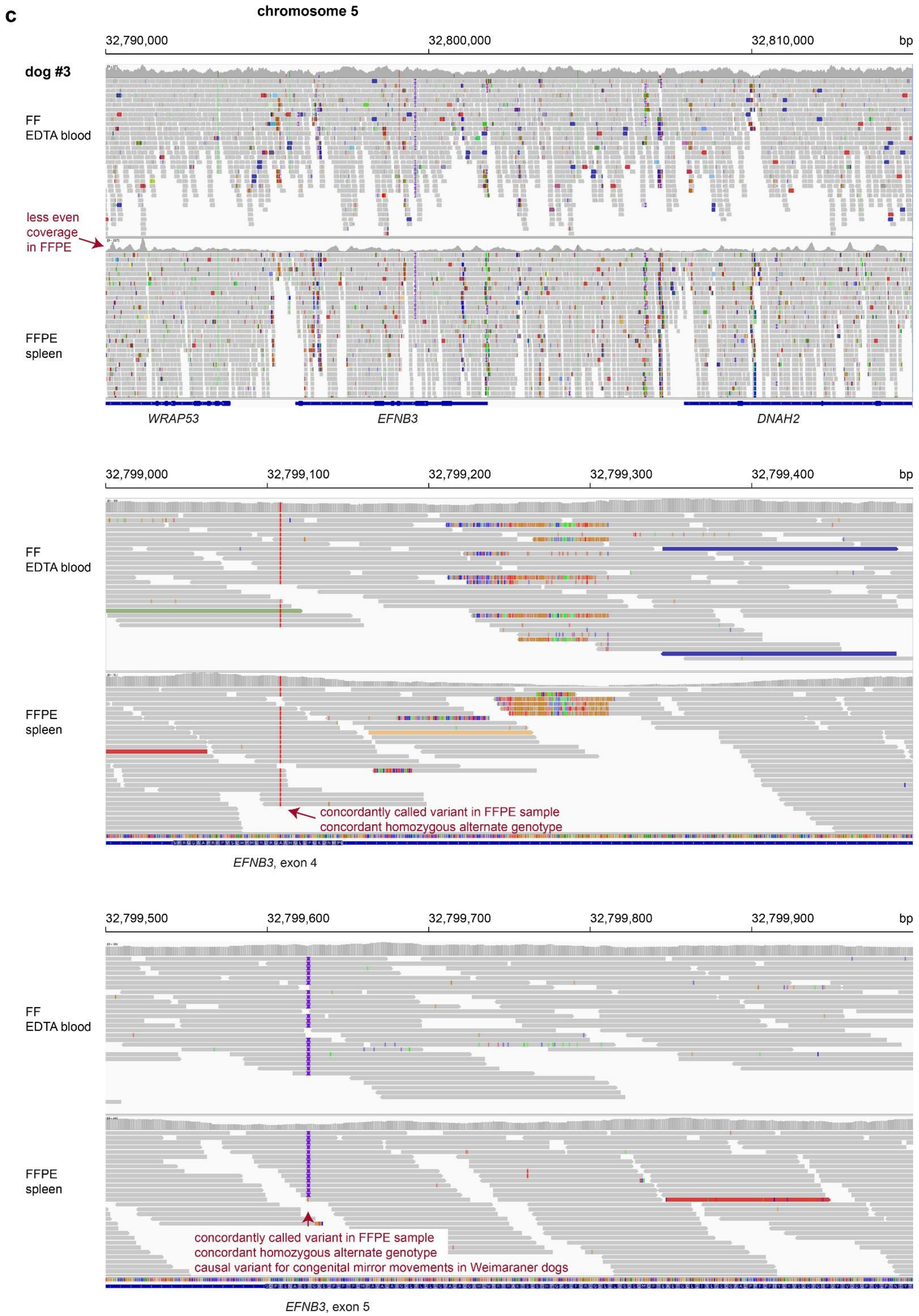
c
chromosome 5
32,790,000
32,800,000
32,810,000
bp
dog #3
FF
EDTA blood
less even
coverage
in FFPE
FFPE
spleen
WRAP53
EFNB3
DNAH2
32,799,000
32,799,100
32,799,200
32,799,300
32,799,400
bp
FF
EDTA blood
FFPE
spleen
concordantly called variant in FFPE sample
concordant homozygous alternate genotype
EFNB3, exon 4
32,799,500
32,799,600
32,799,700
32,799,800
32,799,900
bp
FF
EDTA blood
FFPE
spleen
concordantly called variant in FFPE sample
concordant homozygous alternate genotype
causal variant for congenital mirror movements in Weimaraner dogs
EFNB3, exon 5

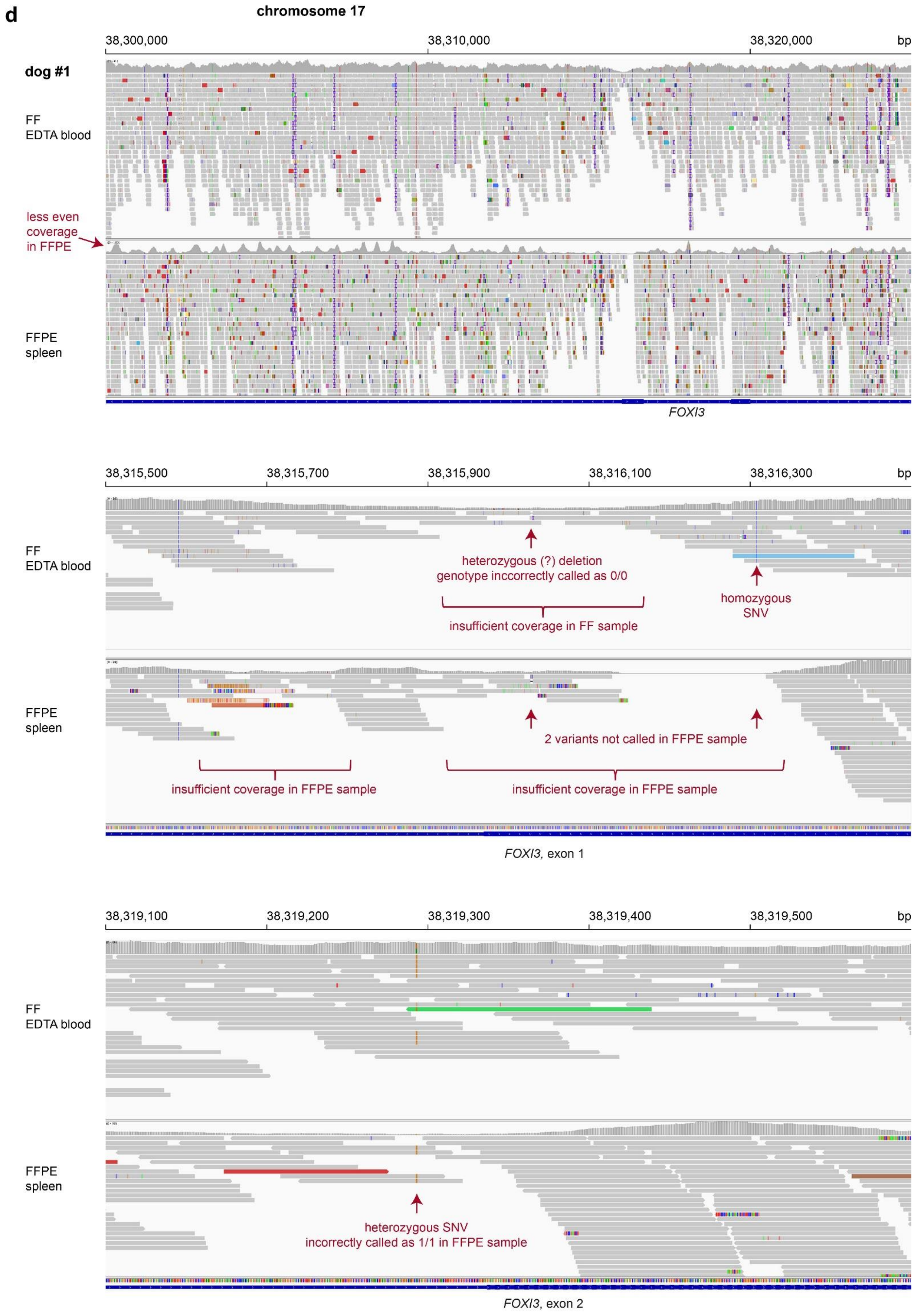
d
chromosome 17
38,300,000
38,310,000
38,320,000
bp
dog #1
FF
EDTA blood
less even
coverage
in FFPE
FFPE
spleen
FOXI3
38,315,500
38,315,700
38,315,900
38,316,100
38,316,300
bp
FF
EDTA blood
heterozygous (?) deletion
genotype inccorrectly called as 0/0
insufficient coverage in FF sample
homozygous
SNV
FFPE
spleen
2 variants not called in FFPE sample
insufficient coverage in FFPE sample
insufficient coverage in FFPE sample
FOXI3, exon 1
38,319,100
38,319,200
38,319,300
38,319,400
38,319,500
bp
FF
EDTA blood
FFPE
spleen
heterozygous SNV
incorrectly called as 1/1 in FFPE sample
FOXI3, exon 2

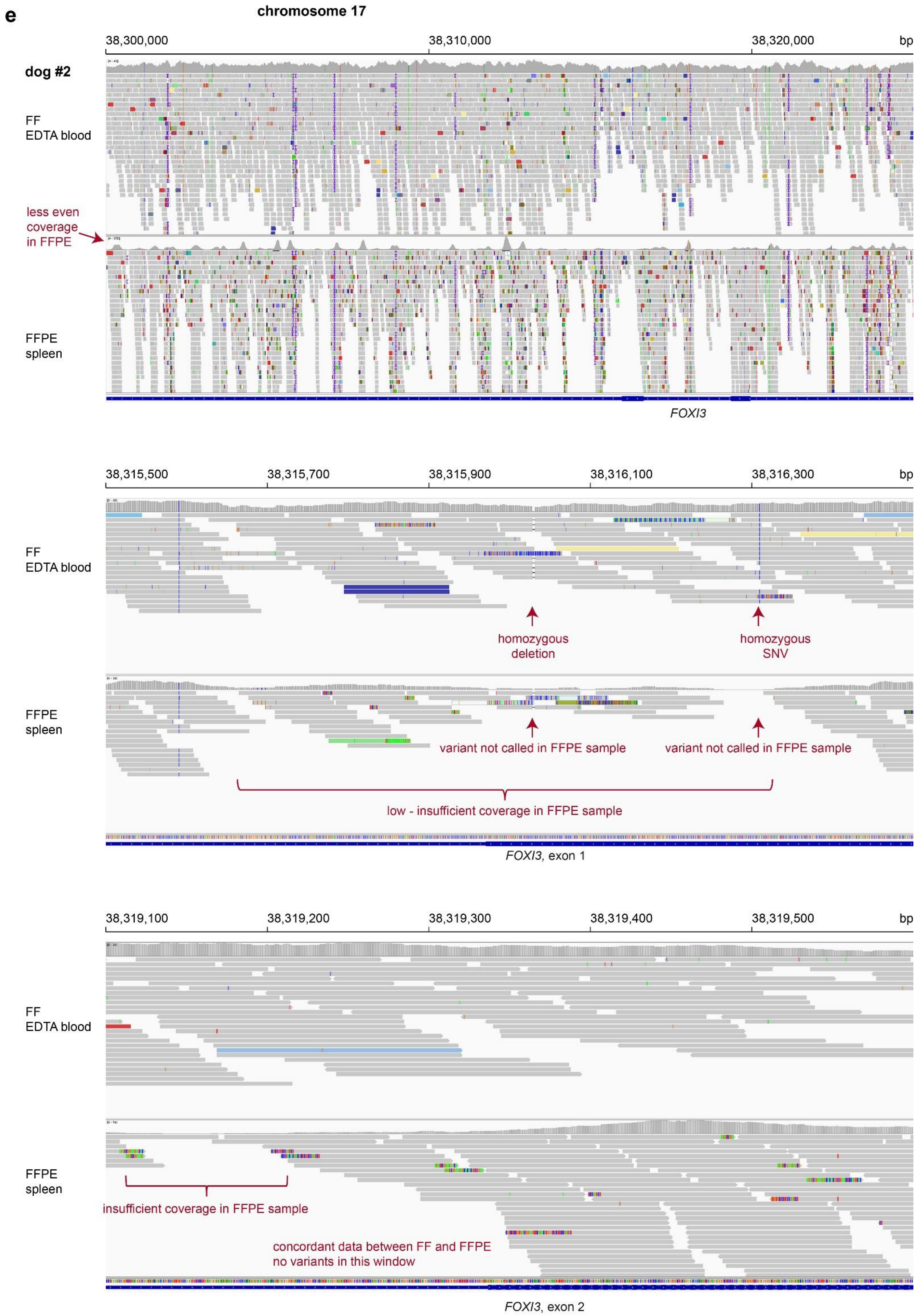

e
chromosome 17
38,300,000
38,310,000
38,320,000
bp
dog #2
FF
EDTA blood
less even
coverage
in FFPE
FFPE
spleen
FOXI3
38,315,500
38,315,700
38,315,900
38,316,100
38,316,300
bp
FF
EDTA blood
homozygous
deletion
homozygous
SNV
FFPE
spleen
variant not called in FFPE sample
variant not called in FFPE sample
low - insufficient coverage in FFPE sample
FOXI3, exon 1
38,319,100
38,319,200
38,319,300
38,319,400
38,319,500
bp
FF
EDTA blood
FFPE
spleen
insufficient coverage in FFPE sample
concordant data between FF and FFPE
no variants in this window
FOXI3, exon 2

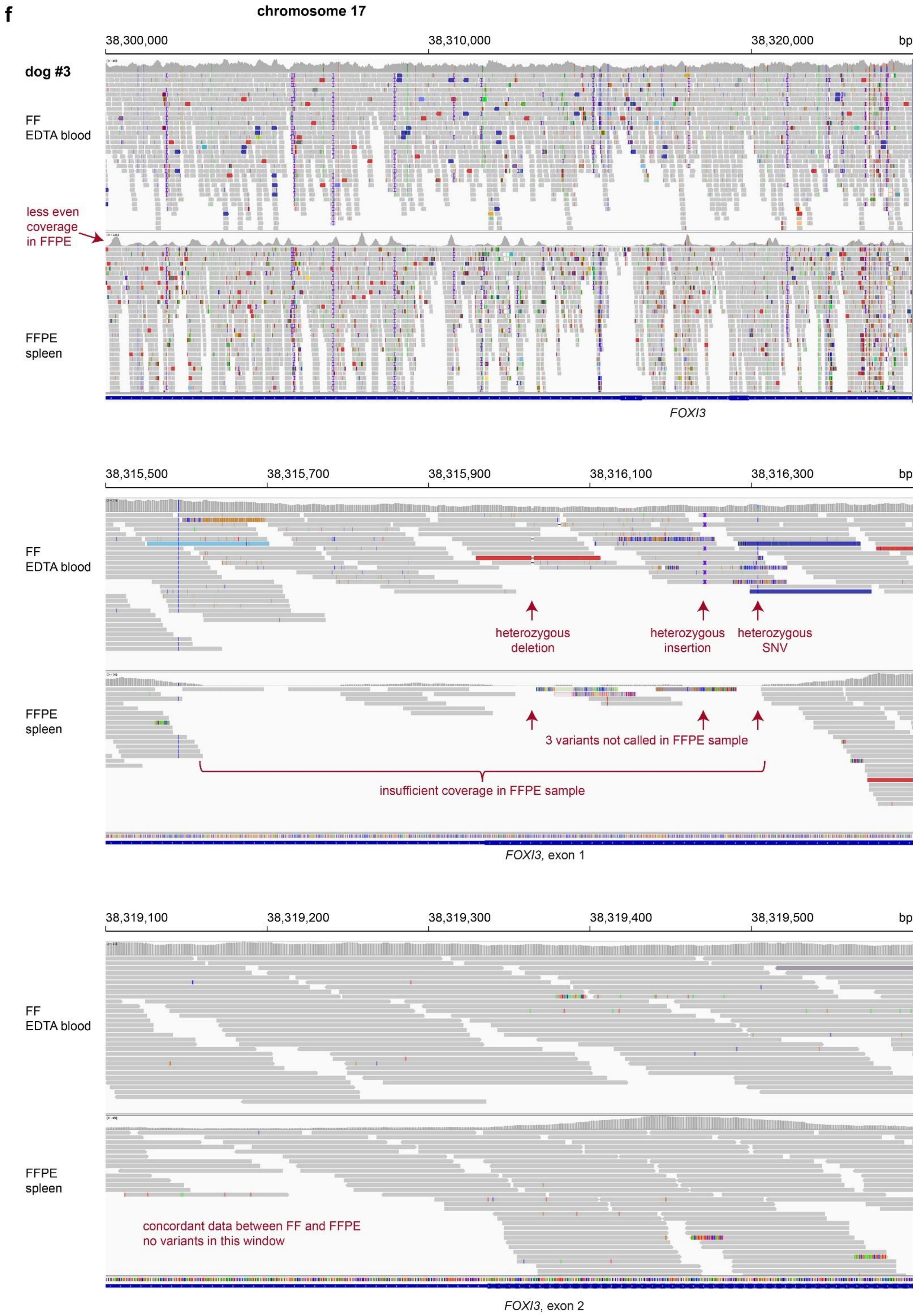
f
chromosome 17
38,300,000
38,310,000
38,320,000
bp
dog #3
FF
EDTA blood
less even
coverage
in FFPE
FFPE
spleen
FOXI3
38,315,500
38,315,700
38,315,900
38,316,100
38,316,300
bp
FF
EDTA blood
heterozygous
deletion
heterozygous
insertion
heterozygous
SNV
FFPE
spleen
3 variants not called in FFPE sample
insufficient coverage in FFPE sample
FOXI3, exon 1
38,319,100
38,319,200
38,319,300
38,319,400
38,319,500
bp
FF
EDTA blood
FFPE
spleen
concordant data between FF and FFPE
no variants in this window
FOXI3, exon 2
